# Cocaine-associated hemoperitoneum following atraumatic splenic rupture: a case report and literature review

**DOI:** 10.1186/1749-7922-8-33

**Published:** 2013-08-28

**Authors:** Faris Azar, Elisha Brownson, Tracey Dechert

**Affiliations:** 1 Department of Surgery, Boston University Medical Center, 850 Harrison Avenue Dowling 2 South, Boston, MA 02118, USA

**Keywords:** Cocaine, Atraumatic splenic rupture, Hemoperitoneum

## Abstract

**Introduction:**

Splenic hematoma or rupture of the spleen is rare in the absence of trauma. This case report with a brief review of the literature is intended to raise awareness of splenic bleeding as an etiology of abdominal pain; it highlights the importance of a detailed social history.

**Presentation of case:**

This report of an otherwise healthy 42-year old man details hemoperitoneum with splenic rupture as a cause for hemorrhage following cocaine use. The patient was managed non-operatively in the surgical intensive care unit. He did not require transfusion and was discharged home on hospital day four with close follow-up.

**Discussion:**

While splenic pathology associated with cocaine use has been described, this case illustrates a novel report of cocaine-associated splenic hemorrhage. A plausible mechanism is transient vasospasm with subsequent bleeding into the infarcted area.

**Conclusion:**

Although uncommon, atraumatic splenic rupture should be recognized early because it is potentially fatal. This case is the first to describe hemoperitoneum of splenic etiology following cocaine use.

## Background

Hematoma or rupture of the spleen is an uncommon finding in the absence of blunt abdominal trauma [1]. Splenic hemorrhage without trauma has been described in pathologic cases, such as infection, but remains exceeding rare in healthy individuals with a normal spleen. Cocaine-associated splenic pathology, ranging from infarction to hematoma, has been previously described in reports in the literature [1-3]. This report of a healthy 42-year old man is the first to describe splenic rupture as a cause for hemorrhage following use of intranasal cocaine. Although uncommon, atraumatic splenic rupture needs to be recognized because it is potentially fatal. This case report with a brief review of the literature is intended to raise awareness of splenic bleeding as an etiology to be included in the differential diagnosis of acute abdominal pain and underlines the importance of a detailed social history.

### Presentation of case

The patient is a 42-year-old man with no significant past medical history, aside from habitual cocaine use, who presented with excruciating left-sided abdominal pain after he consumed intranasal cocaine. The pain was constant, sharp, and nonradiating. Two days prior to presentation, he felt an acute onset of left upper quadrant pain immediately following a cough. The pain then became diffuse and more severe, prompting him to seek treatment in the emergency department (ED). He endorsed a similar left upper quadrant pain a few weeks prior, but that episode was less severe and resolved on its own. He denied any history of trauma, sick contacts, or recent travel.

On arrival to the ED, the patient’s vital signs were as follows: temperature of 36.7 degrees centigrade, blood pressure of 103/68 mm Hg, pulse rate of 100 beats per minute, respiratory rate of 16 breaths per minute, and an oxygen saturation of 97% on room air. On physical examination, a subtle swelling of the left upper quadrant was noted. The abdomen was soft but markedly tender to palpation diffusely with mild guarding.

**Figure 1:**
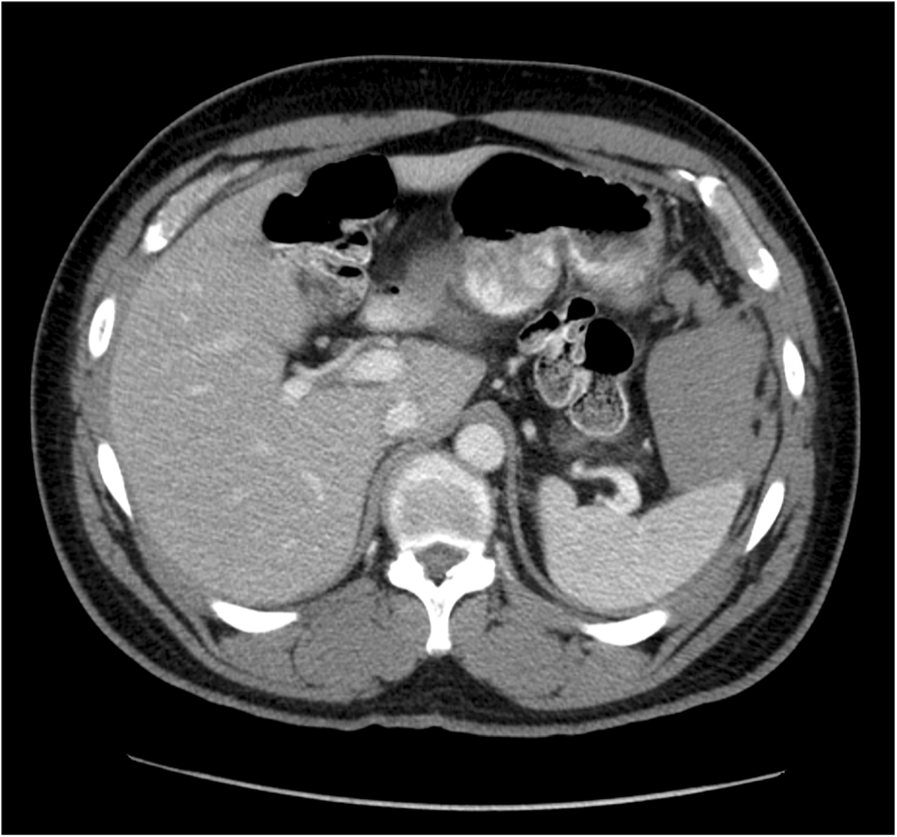
Axial, contrast-enhanced CT image demonstrates moderate hemoperitoneum in left upper quadrant centered around the spleen.

**Figure 2:**
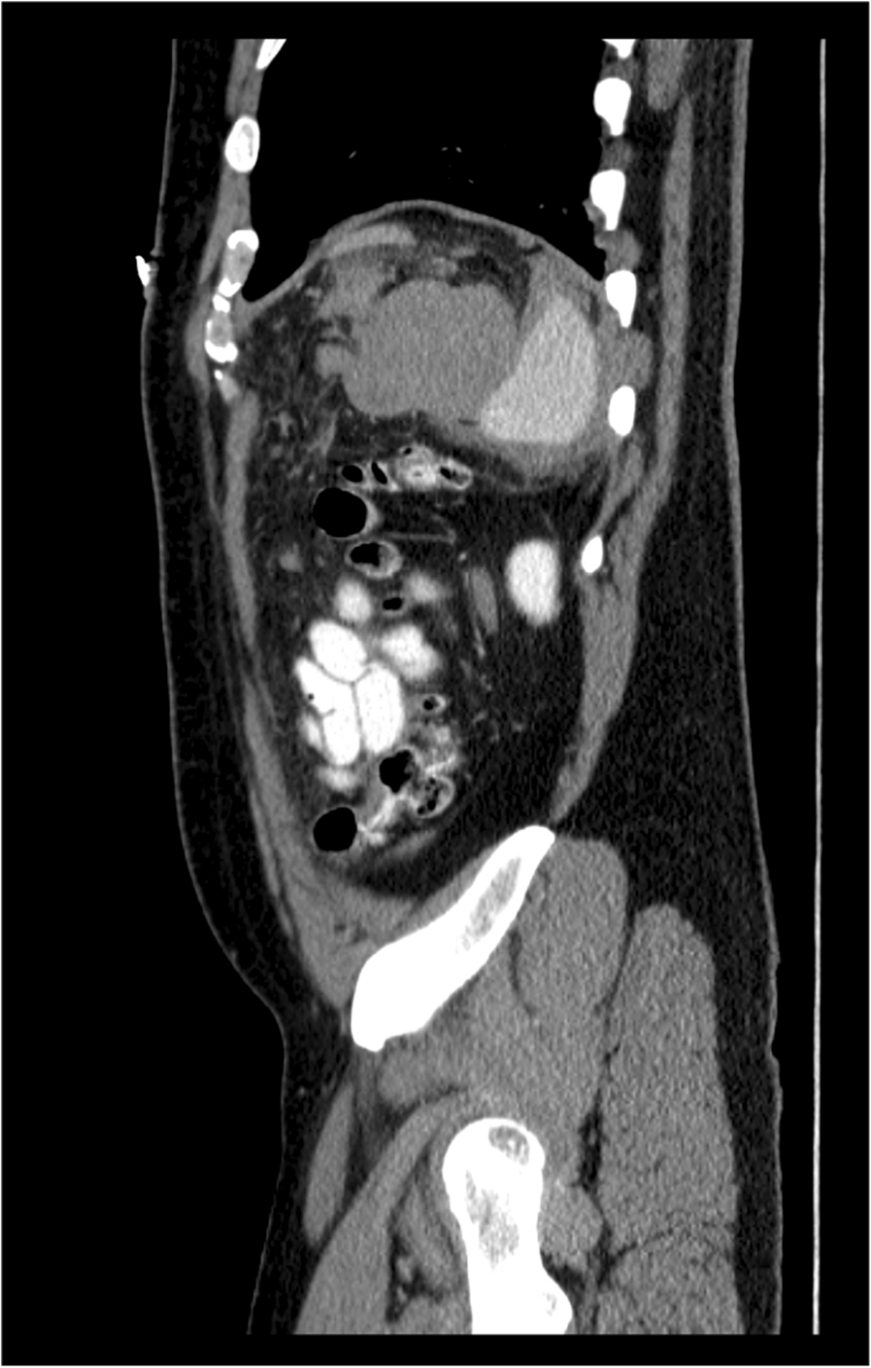
Sagittal, contrast-enhanced CT image demonstrates perisplenic hematoma.

**Figure 3:**
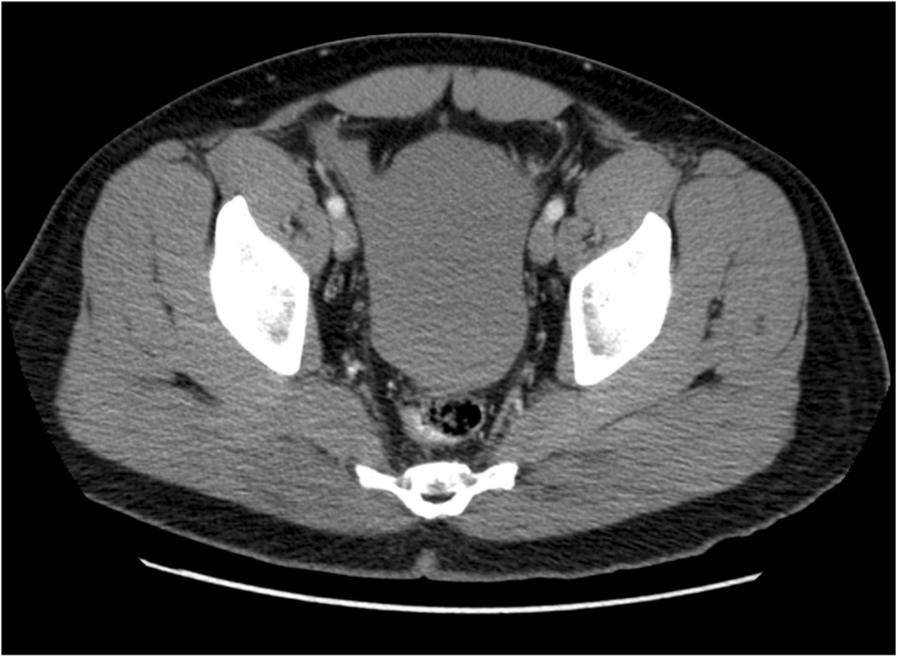
Axial, contrast-enhanced CT image of the pelvis demonstrates large hemoperitoneum.

Laboratory studies revealed an initial hematocrit of 42.8%, and urine toxicology was positive for cocaine. Computed tomography (CT) scan of the abdomen and pelvis with oral and intravenous contrast showed no evidence of free peritoneal air or injury to any solid organs or bones including the ribs, but did reveal fluid around the spleen, in the left paracolic gutter, and layering in the pelvis (Figures 1, 2 and 3). There was no evidence of active contrast extravasation, no vascular blushes or aneurysms, no findings of portal hypertension, and no suspicion for malignancy. These radiographic findings pointed to a splenic source for hemoperitoneum. Six hours after presenting to the ED, the patient’s hematocrit had dropped to 36.6%, and repeat CT scan revealed a focal collection of fluid surrounding the spleen. Given that the patient remained hemodynamically stable, he was admitted for non-operative management in the surgical intensive care unit, where he had serial abdominal examinations and blood count monitoring.

The patient did not require transfusion as his hematocrit remained stable between 36% and 38% throughout his hospital course. During that time, infectious etiologies including Epstein-Barr virus and cytomegalovirus were ruled out as possible causes. A human immunodeficiency virus test performed two weeks prior to this admission was negative. Additionally, hematologic malignancy was excluded with a peripheral blood smear. The patient’s symptoms significantly improved and he was discharged on hospital day four.

**Figure 4:**
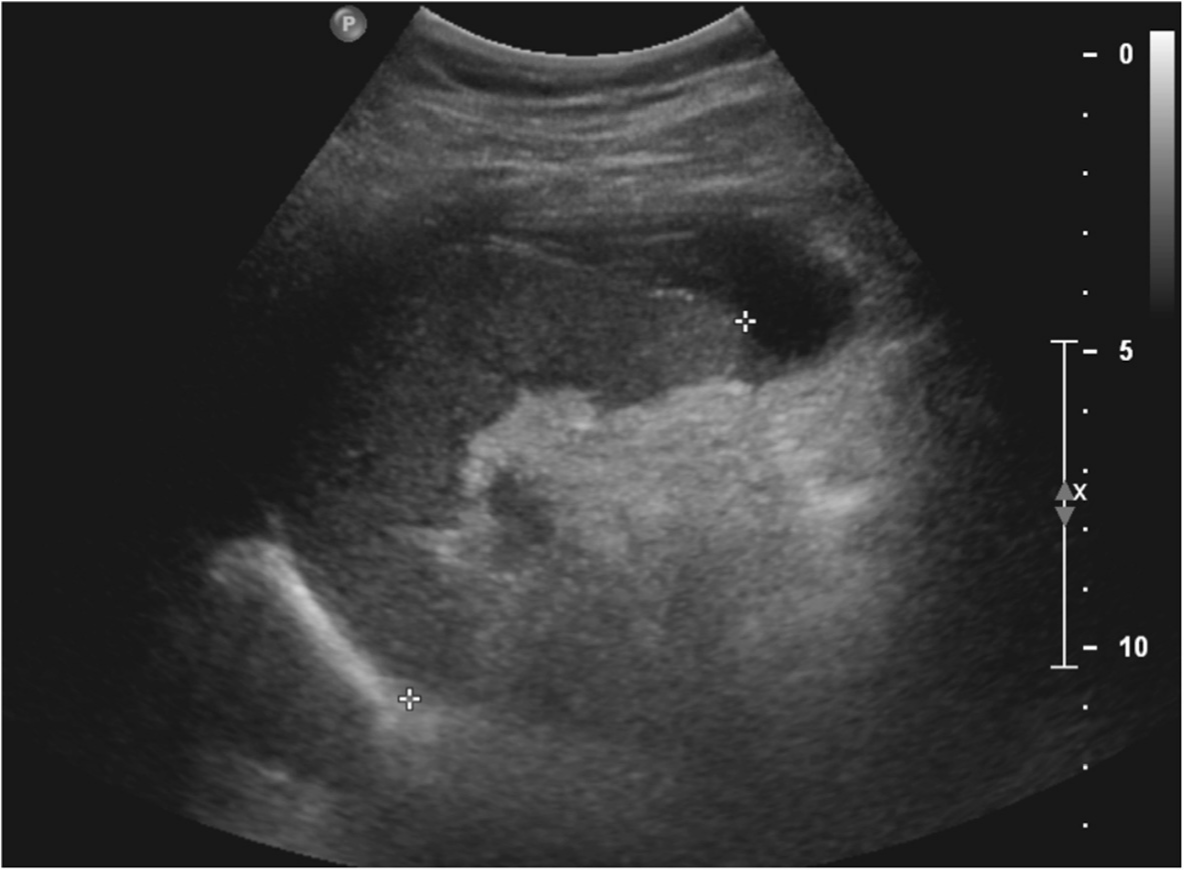
2D gray scale ultrasound image demonstrates small degree of subcapsular splenic hematoma.

On follow-up ten days after initial presentation, the patient’s symptoms had resolved and his vital signs were stable. An abdominal ultrasound revealed a subcapsular splenic hematoma at the tip of the spleen tracking anteriorly with interim resolution of free fluid in the pelvis, confirming a splenic etiology for hemoperitoneum (Figure 4). Although the patient’s CT scan did not show a blush suggestive of a pseudoaneurysm, the diagnosis of a splenic artery pseudoaneurysm could have been investigated further with a splenic angiogram.

## Conclusions

Splenic rupture in the absence of trauma is exceedingly rare. Although atraumatic splenic rupture (ASR) is uncommon, it warrants early recognition due to the potentially fatal consequences and thus should be included in the differential diagnosis of patients with left upper quadrant abdominal pain [2]. A recent systematic review of atraumatic splenic rupture found there to be six major etiological groups: neoplastic processes (30.3%), infectious (27.3%), inflammatory (20.0%), iatrogenic (9.2%), mechanical (6.8%), and normal spleen (6.4%) [1]. ASR of the normal spleen is defined by four criteria: no history of trauma, no evidence of extrasplenic disease known to affect the spleen, no perisplenic adhesions to suggest previous trauma, and normal spleen on gross and histologic exam [3].

Clinical presentation of ASR mimics traumatic splenic rupture. Abdominal pain, especially in the left upper quadrant, or chest pain with radiation to the left shoulder, caused by subdiaphragmatic irritation, are classic symptoms of splenic pathology. There is often little or no clinical history to suggest splenic pathology, and the diagnosis is often made after imaging, which often includes ultrasonography or CT scan [4].

There are no definitive guidelines on management of ASR, although it is often modeled after that of traumatic splenic rupture. Treatment may include operative or non-operative therapy, depending upon the patient’s hemodynamic stability and degree of splenic injury. The large amount of fluid within the abdomen could support operative evaluation with exploratory laparotomy. Factors favoring non-operative management in this case included total clinical stability, a soft abdomen, and duration of greater than 24 hours from the inciting event. The American Association for the Surgery of Trauma criteria for degree of splenic injury correlates with failure of conservative treatment. Given that a splenic etiology was not confirmed until the ultrasound after discharge, his injury could not be graded. At the time of follow-up, the subcapsular hematoma measured less than 10% of the surface area, consistent with a grade 1 injury [5]. Even in the setting of non-operative management, surgical teams are often involved or are the primary team managing inpatient surveillance. Work-up in patients with ASR should include studies to rule out the common causes, including neoplastic, infectious, and inflammatory processes. As this patient’s work-up was negative, we conclude that the patient had a normal spleen with ASR and associate the splenic rupture with cocaine use.

Cocaine use remains epidemic and is associated with a wide range of medical complications. The well-studied physiologic effects of cocaine include increased norepinephrine reuptake with sustained alpha-adrenergic receptor stimulation and resultant vasoconstriction. Cocaine-associated vasoconstriction was shown to transiently reduce splenic volume on average by 20% [6]. This vasoconstriction transiently elevates blood pressure. In addition, increased abdominal venous pressure due to cough could suggest an inciting event for splenic hemorrhage in this patient. This has been previously described in a case report of a patient with hemoperitoneum after ingestion of cocaine and associated acute emesis; however, no etiologic source or evidence of underlying pathology was found [7].

Splenic infarction following cocaine use is rare but has been described, particularly in patients with sickle hemoglobinopathies [8]. It is plausible that cocaine-associated splenic hematoma or rupture results from transient vasospasm with subsequent bleeding into the infarcted area. Secondary infection of the infarcted spleen with resultant sepsis and death has also been detailed [9].

While the use of cocaine causing hematoma of the spleen has been described [10], this case is the first report of a case that details hemoperitoneum caused by ASR following cocaine use. Although uncommon, the potential for death due to splenic rupture warrants awareness and highlights the importance of a social history in patients presenting with acute abdominal pain.

### Consent

Written informed consent was obtained from the patient for publication of this Case report and any accompanying images. A copy of the written consent is available for review by the Editor-in-Chief of this journal.

## Abbreviations

ED: Emergency department; CT: Computed tomography; ASR: Atraumatic splenic rupture.

## Competing interests

The authors declare that they have no competing interests.

## Authors’ contributions

FA and EB conducted the literature search and completed the chart review. FA authored the manuscript. EB edited the manuscript. EB provided patient care. TD was the attending physician who cared for the patient, instigated the study, edited the manuscript, and oversaw the project. All authors read and approved the final manuscript.

## References

[B1] Renzulli P Hostettler A Schoepfer AM Gloor B Candinas D Systematic review of atraumatic splenic rupture Br J Surg 2009 8 10 1114 1121 10.1002/bjs.6737 19787754

[B2] Wehbe E Raffi S Osborne D Spontaneous splenic rupture precipitated by cough: a case report and a review of the literature Scand J Gastroenterol 2008 8 5 634 637 10.1080/00365520701763472 18415760

[B3] Debnath D Valerio D Atraumatic rupture of the spleen in adults J R Coll Surg Edinb 2002 8 437 445 11874265

[B4] Amonkar SJ Kumar EN Spontaneous rupture of the spleen: three case reports and causative processes for the radiologist to consider Br J Radiol 2009 8 e111 e113 10.1259/bjr/81440206 19451309

[B5] Tinkoff G Esposito TJ Reed J Kilgo P Fildes J Pasquale M Meredith JW American Association for the Surgery of Trauma Organ Injury Scale I: spleen, liver, and kidney, validation based on the National Trauma Data Bank J Am Coll Surg 2008 8 5 646 10.1016/j.jamcollsurg.2008.06.342 18954775

[B6] Kaufman MJ Siegel AJ Mendelson JH Rose SL Kukes TJ Sholar MB Cocaine administration induces human splenic constriction and altered hematologic parameters J Appl Physiol 1998 8 5 1877 1883 9804594 10.1152/jappl.1998.85.5.1877

[B7] Bellows CF Raafat AM The surgical abdomen associated with cocaine abuse J Emerg Med 2002 8 4 383 386 10.1016/S0736-4679(02)00576-0 12480020

[B8] Vaghjimal A Splenic infarction related to cocaine use Postgrad Med J 1996 8 854 768 9015479 10.1136/pgmj.72.854.768-b PMC2398657

[B9] Dettmeyer R Schlamann M Madea B Cocaine-associated abscesses with lethal sepsis after splenic infarction in an 17-year-old woman Forensic Sci Int 2004 8 1 21 23 10.1016/j.forsciint.2003.11.031 15013162

[B10] Homler HJ Nontraumatic splenic hematoma related to cocaine abuse West J Med 1995 8 2 160 162 7571571 PMC1303019

